# A mechano-sensing mechanism for waving in plant roots

**DOI:** 10.1038/s41598-022-14093-1

**Published:** 2022-06-10

**Authors:** Zhenwei Zhang, Danie van Ophem, Raghunath Chelakkot, Naftali Lazarovitch, Ido Regev

**Affiliations:** 1 French Associates Institutes for Agriculture and Biotechnology, Jacob Blaustein Institutes for Desert Research, Ben-Gurion University of the Negev, Sede Boqer Campus, 84990 Israel; 2 Department of Physics, Indian Institute of Technology Bombay, Mumbai, 400076 India; 3 Department of Solar Energy and Environmental Physics, Jacob Blaustein Institutes for Desert Research, Ben-Gurion University of the Negev, Sede Boqer Campus, 84990 Israel

**Keywords:** Plant physiology, Applied mathematics, Biological physics, Nonlinear phenomena

## Abstract

Arabidopsis roots grown on inclined agar surfaces exhibit unusual sinusoidal patterns known as root-waving. The origin of these patterns has been ascribed to both genetic and environmental factors. Here we propose a mechano-sensing model for root-waving, based on a combination of friction induced by gravitropism, the elasticity of the root and the anchoring of the root to the agar by thin hairs, and demonstrate its relevance to previously obtained experimental results. We further test the applicability of this model by performing experiments in which we measure the effect of gradually changing the inclination angles of the agar surfaces on the wavelength and other properties of the growing roots. We find that the observed dynamics is different than the dynamics reported in previous works, but that it can still be explained using the same mechano-sensing considerations. This is supported by the fact that a scaling relation derived from the model describes the observed dependence of the wavelength on the tilt angle for a large range of angles. We also compare the prevalence of waving in different plant species and show that it depends on root thickness as predicted by the model. The results indicate that waving can be explained using mechanics and gravitropism alone and that mechanics may play a greater role in root growth and form than was previously considered.

## Introduction

One of the outstanding problems in plant science is understanding the role played by the environment in plant growth^1^. It is well known that plants respond to variations in environmental conditions by changing the direction of growth^2^. Examples for environmental cues that are detected by plants are moisture (hydrotropism), light (phototropism), salinity (halotropism) and gravity (gravitropism)^3–6^. In some cases, the mechanism that allows the plant to detect these cues at the cellular level is known. For example, it is known that the direction of gravity is detected by specific cells called Statoliths^7^ and there are suggestions as to how these cells detect the direction of gravity^8^. It is also known that plants respond to mechanical obstacles (thigmotropism)^9^ which raises the possibility that root tips can sense pressure^10^. However, there is currently no clear candidate for an equivalent mechanism for pressure sensitivity (there are some suggestions for such mechanisms, based on the response of cell wall growth to pressure^11^ but these are not fully confirmed). This raises the possibility that a plant’s response to mechanical obstacles can be, at least in some cases, attributed to a ‘passive’ feedback between the mechanics of the plant and its environment and the growth of the plant rather than to ‘active’ thigmotropism.

**Figure 1:**
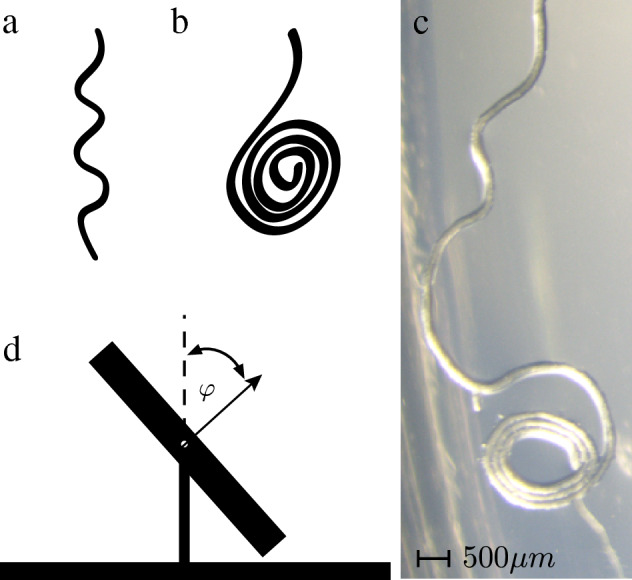
Root waving and coiling: (**a**) An illustration of root waving. (**b**) An illustration of root coiling. (**c**) An image showing an Arabidobsis root transitioning from a waving pattern to a coiling pattern. (**d**) An illustration of the experimental apparatus containing an agar-covered plastic plate (petri-dish) the normal of which can be set at different angles $\varphi$ with respect to the vertical. The plate is shown from the side.

One phenomenon in which such a mechanism has been suggested is root waving in species such as *Arabidopsis thaliana*. When grown on a plastic plate (a petri-dish) containing an agar gel tilted at an angle of $45^\circ$ with respect to the vertical, the roots of these plants grow in a sinusoidal form^12^. A related phenomenon occurs when the plate is laid horizontally—under these conditions roots grow in concentric circles^12^ (see Fig. 1a–c). While it is generally accepted that waving and coiling are results of the resistance of the substrate to the gravitropic tendency of the root to grow vertically, it is not known if the sine and circular patterns are a result of a purely biological mechanism such as circumnutation or auxin expression, or if they are related to a mechanical response of the roots to the forces generated by growth and substrate resistance. Circumnutation is a circular motion of growing shoots, which was recently also observed in rice roots^13^. There are suggestions that waving is some form of projection of this growth pattern into two-dimensions^14,15^. Auxin is a plant growth hormone that was shown to play a role in the periodic branching of roots and was also suggested as a possible mechanism for waving^15–17^ . It is also possible that mechanical and biological factors act simultaneously to determine the dynamical behaviour of growing roots.

Thompson and Holbrook^18^ have noticed that the root tip indents the surface of the gel, due to the gravitropic force, which they proposed, increases the friction at the root tip. This, they suggest, causes the root tip to move slower with respect to the rest of the root, which, together with the fact that most of the root is anchored by root hairs, while the part close to the root tip is free to move, causes the part that is not anchored to bend and grow sideways. In their conceptual model, the friction is kinetic and its effect is to amplify existing curvature or curvature fluctuations. They also suggested that the periodic change in curvature associated with waving is induced by gravitropism.

Here we suggest a semi-quantitative model in which the friction is dry which, we believe, is a more reasonable assumption in light of the fact that the contact between the root and the agar plate is taking place in the open air. This assumption naturally leads to a stick-slip type motion—the root tip is held by static friction most of the time and it progresses in short slip bursts when the growth overcomes the friction. In this model, curving of the root occurs due to mechanical buckling that ensues when the part of the root that is not held by root hairs is long enough. The buckling explains why the root tip changes the growing direction and thus why waving occurs. We test this hypothesis by comparing the analytical solution of the buckling equations to snapshots of root waving dynamics obtained by Thompson and Holbrook^18^.

We follow by quantifying the directions at which forces are applied by the root tip, and conclude that they can be decomposed into a component pointing inside the agar gel, leading to a normal force and friction, and components that direct the growth to the downhill direction. The latter, we suggest, breaks the symmetry and allows for waving rather than coiling when the agar plate is tilted but not horizontal.

We next study the effect of changing the angle experimentally. We observe changes in wavelength, amplitude and in switching between waving and coiling as a result of changes in the tilt angle. However, contrary to what was observed in the experiments by Thompson and Holbrook, in our experiments the wavelength is much larger. We explain qualitatively how the same mechanism can also explain the dynamics observed in our experiments and show that the change in wavelength as a function of tilt angle can be described using dimensional considerations based on the suggested mechanics.

We also measure the probability of a root to coil as a function of the tilt angle from our experimental data and observe that circular growth occurs not only when the plate is completely horizontal but also at smaller angles (since we measure the angle $\varphi$ of the normal to the surface with respect to the vertical direction, $\varphi =90^{\circ }$ is the angle when the plate is vertical and $\varphi =0^{\circ }$ is the angle when the plate is horizontal). However, the exact angle at which a root switches to circular growth is random. This suggests that the transition from waving to circular growth is a bifurcation but that due to natural variability in the plants and growing medium, the bifurcation point becomes smeared.

## A model for growth with friction

It is known that when grown on agar surfaces, roots anchor to the surface using thin hairs which come out of the main root. However, the hairs start to grow at some distance from the tip (in Arabidopsis this distance is about $1$ mm) which means that the part of the root close to the root tip is free to move with respect to surface. In experiments studying the dynamics of root waving^18^, it was noticed that root waving occurs due to bending of this part of the root, which is not tethered to the gel by the root-hairs, and it was suggested that this bending is related to friction that is felt by the root-tip due to its tendency to indent the surface of the gel. In the following we will call this part of the root, which includes the root tip and the growth zone (a region close to the root tip where cells divide and grow), the Freely-Moving-Zone (FMZ).

Since the root hairs grow at a constant rate, the average length of the FMZ does not change during growth. However, from the images by Thompson et al.^18^, we observe that the root hairs are separated by $\approx 200\, \upmu$m from each other on the same side of the root and by $\approx 100 \,\upmu$m on opposite sides whereas the length of the FMZ is $\approx 1250 \,\upmu$m. Therefore, the length of the FMZ is somewhat variable and is oscillating between $\approx 1200 \,\upmu$m and $\approx 1300\,\upmu$m. This indicates that the variation in the length of the FMZ cannot be neglected and growth can play a significant role in the dynamics. We therefore suggest a mechanical model for growth dynamics following the general ideas put forward by Thompson and Holbrook^18^, combining of gravitropically induced friction and the effects of growth of the FMZ itself. The model describes and explains aspects of the three different forms of growth—straight growth, waving and coiling. The specific pattern formed depends on physical properties of the root and the orientation of the plane on which the growth occurs.

### Stick-grow-slip

In the following we assume that growth occurs when the root tip is pinned by static friction and that the periods in which the root tip slips are very short and occur when the stress accumulated due to growth and confinement allows the tip to overcome the static friction. The logic behind this assumption is that growth is a very slow process while sliding and elastic deformation are very fast processes. While hydrogels such as agar are wet materials, and can exhibit hydrodynamic lubrication at high velocities, it is found experimentally that at low sliding velocities they exhibit static friction and stick slip dynamics, due to some form of adhesion^19,20^ which further justifies our assumptions.

We argue that the elastic response of the FMZ to the mechanical load determines the deformation mode. We will first describe the simplest case—straight growth. This kind of growth occurs in some roots which have relatively thick roots (e.g. tomato) and do not show waving or coiling. This growth mode can be separated into two parts: *Slow growth with pinning*—in which the actual length of the FMZ increases due to growth but the root tip does not move due to static friction. In this stage the axial load acting on the FMZ (which we view as a cylindrical rod of radius *r*) increases due to the incompatibility of the grown FMZ with the boundary conditions imposed by the root hairs and friction. *Fast slip*—once the force at the root tip becomes larger than the maximal static friction, the root tip starts to move in a fast slip which is quickly halted due to dynamic friction.

**Figure 2:**
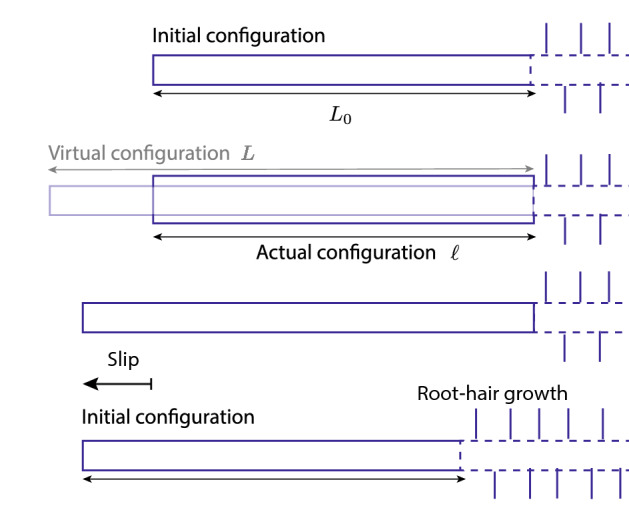
Different stages during stick-grow-slip without buckling. From top to bottom: Initial configuration, virtual (grown) configuration vs the actual configuration, stress release by slip, new initial configuration.

To model growth under confinement and the resulting forces, we follow Goriely^21^ and model the growth using three “configurations”. The initial configuration, before the growth starts, has a length $L_0$. Once growth has started, this configuration changes into a “virtual” configuration of length $L = \gamma L_0$ where $\gamma > 1$ (see Supplementary Material for a more precise definition of $\gamma$). Since the FMZ is clamped and pinned in both ends, the virtual configuration will be elastically deformed by a factor $\alpha$ to form the current (actual) configuration and its length *L* will be scaled by $\alpha <1$ such that $\ell = \alpha L$. The total deformation factor $\uplambda$ is thus a multiplication of the growth rate $\gamma$ and the elastic deformation $\alpha$:

1 $$\begin{aligned} \uplambda = \gamma \alpha , \end{aligned}$$

such that $\ell = \uplambda L_0$. If the boundaries are fixed and the FMZ is straight (not buckled) the deformation factor is constant $\uplambda =1$ and the elastic deformation $\alpha = 1/\gamma$ increases due to the increase in $\gamma$ (growth). For a growth rate $d\gamma /dt = k$, this causes the accumulation of mechanical stress described by:

2 $$\begin{aligned} \sigma = E(\alpha - 1) = -E\left( \frac{kt}{1+kt}\right), \end{aligned}$$

(here *x* is the horizontal direction and *E* is Young’s modulus of the root) and the appearance of an axial force (note that this force is negative since the grown FMZ is compressed in order to fit the boundary conditions). The axial force, that is identical to the force applied by the tip, is then:

3 $$\begin{aligned} F_x = -A\sigma = AE\left( \frac{kt}{1+kt}\right), \end{aligned}$$

which acts against the static friction. Here $A = \pi r^2$ is the root cross section area. This force is balanced by the static friction $f_s$ as long as:

4 $$\begin{aligned} f_s < \mu _s F_N, \end{aligned}$$

where $\mu _s$ is the static friction coefficient and $F_N$ is the normal force. Once this threshold is reached, the root tip slips and the friction becomes kinetic, that causes the elastic energy to dissipate and which eventually brings the root tip to a stop (for details refer to Supplementary Material). The root is then confined again by static friction, continues to grow, while the root hairs also grow bringing the FMZ back to its initial length. An illustration of the different stages of the dynamics is shown in Fig. 2 and an approximate calculation of stick and slip times, forces and slip length can be found in the Supplementary Material.

We would also like to stress that stick-grow-slip dynamics was not yet observed directly since the slip events are expected to be very short. In the Supplementary Material we estimate the stick and slip times but can only give approximate estimates since some of the constants are unknown. Therefore, observing how slip occurs in very short times is a goal for further research. For plants with thick roots (for example, the roots of tomato), slipping without buckling is the only mode of propagation. However, in plants with thin roots, such as *Arabidopsis thaliana*, the stick and growth stage is accompanied by bending that as will be explained below, originates in mechanical buckling.

**Figure 3:**
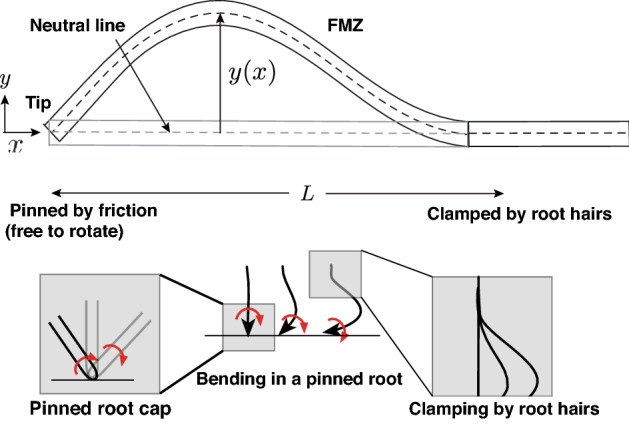
Geometry and boundary conditions: The freely moving zone is modeled as a thin elastic rod subject to a compressive force which arises from the combination of static friction at the tip and growth. On the right, the rod is clamped due to anchoring by root-hairs—it cannot change its position *and* its angle. On the left the rod is pinned—it can change direction but cannot move. For a large enough compressive force, the FMZ buckles, which allows for growth in the lateral direction, which is assumed to be on the surface of the agar, as is observed in the experiments. In the figure below, we show a possible mechanism for twisting of the FMZ.

### Stick-grow-bend-slip

As was mentioned above, we model the FMZ as an elastic rod with a cylindrical profile of radius *r*, subject to an axial force $F_x$, given by Eq. (3), that increases as a result of the combination of growth and confinement. For a long and thin FMZ, the increase in the axial force $F_x$, can cause it to buckle and bend, which allows for a much larger root elongation during the “stick” phase. While bending into the growth plane does occur due to gravitropism, buckling in the direction normal to the surface is not observed in most cases and the buckling that is observed is almost always on the surface of the agar plate^18^. A possible reason is that due to the pinning, the root tip is unstable to rotations, and when the FMZ is out of plane, it rotates until the plane that includes the FMZ becomes parallel to the agar plate. In Fig. 3 we show a schematic of the FMZ before and after buckling, where the x-axis is defined to be along the direction of the FMZ before buckling and the y-axis is the transverse direction—both are taken to be on the surface of the agar. These conventions will be used throughout this sub-section.

For this growth mode to become available, the force $F_B$ at which buckling occurs must be smaller than the maximal static friction force $f_s = \mu _sF_N$. For an FMZ of length *L* and bending modulus *B*, that is anchored to the gel due to the root hairs on one end, and pinned due to friction on the other end, (clamped-pinned boundary conditions) the force $F_B$ at which the rod buckles can be calculated analytically^22^:

5 $$\begin{aligned} F_B = \frac{\beta ^2 B}{L^2}, \end{aligned}$$

where:

6 $$\begin{aligned} \beta \approx 4.49341, \end{aligned}$$

is a dimensionless number^23^. If the maximal static friction is smaller than $F_B$:

7 $$\begin{aligned} \mu _s F_N < \frac{\beta ^2 B}{L^2}, \end{aligned}$$

buckling will not occur, and growth will continue in the manner described in the previous section. As we will discuss below, this happens in some plants. However, if *L* is large enough and *B* is small enough (this typically happens for thin roots since $B\sim r^4$) such that $F_B$ is smaller than the maximal friction, the FMZ will buckle and the rod will start bending.

**Figure 4:**
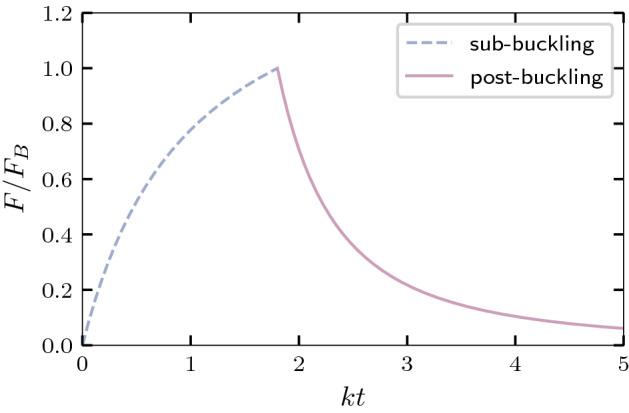
Post-buckling: The force $F = \sqrt{F_x^2 + F_y^2}$ (rescaled by the buckling force $F_B$) before (dashed blue line) and after buckling occurs as a function of *kt*, without taking into account root hair progression.

When a rod of fixed length buckles, the axial force increases as a function of the deflection. Therefore, we expect the root tip to overcome the frictional force shortly after the buckling event. However, as we show in the Supplementary Material, by adding growth to the perturbative solution of Wang^23^, the post-buckling force at the root tip is found to be:

8 $$\begin{aligned} F \approx F_B\frac{1}{(1 + k(t-t_b))^2}\left[ 1 + \frac{{\mathcal {C}}}{\beta ^2}\left( \frac{k(t-t_b)}{1 + k(t-t_b)}\right) \right] , \end{aligned}$$

where ${\mathcal {C}}$ is a constant and $t_b$ is the time at which buckling occurs, assuming that growth started at $t=0$. In Fig. 4, we can see that this is a decreasing function of time, which means that for a growing rod, subject to the aforementioned boundary conditions, it is mechanically favorable to keep growing even though the curvature is increasing. As one can observe in the movies by Thompson and Holbrook^18^, where the root hairs are visible, it is clear that the continual addition of root hairs eventually causes the curvature of the FMZ to stop increasing. In their experiments, once the root hairs reach the point of maximal deflection (in some cases even before that), the FMZ effectively returns to its “short” phase and $F_B$ becomes larger than $f_s$ again, which causes the root tip to return temporarily to the stick-grow-slip phase and experience several slip events until *L* reaches the buckling length again.

**Figure 5:**
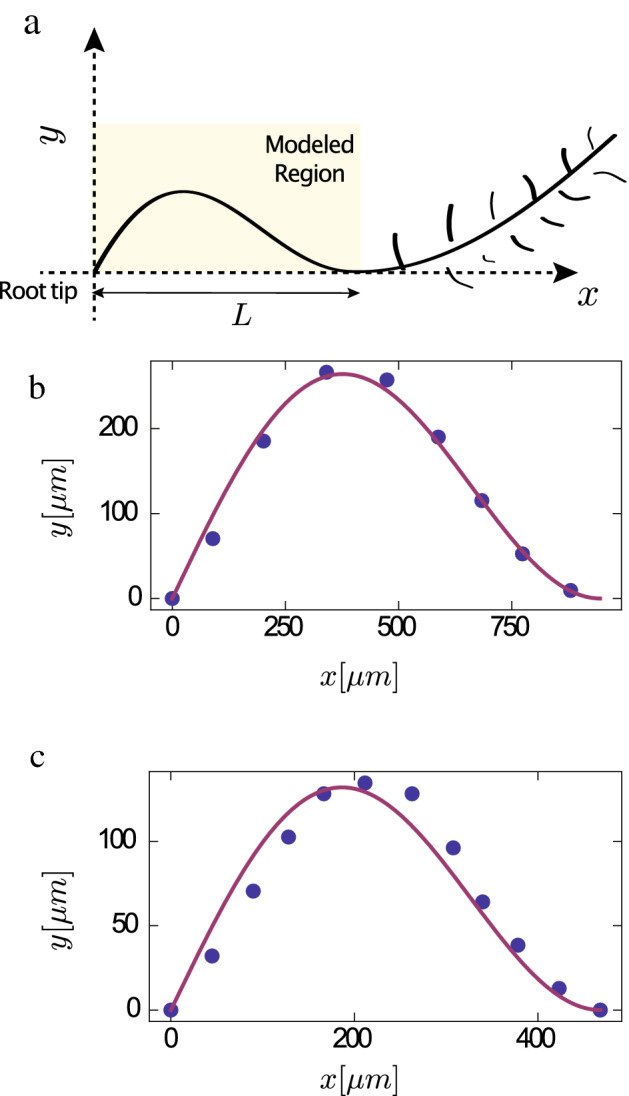
Comparing experiments to theory: (**a**) An illustration of the region of the root in the experiments of Thompson and Holbrook^18^ that is compared to the elastic solution for pinned-clamped boundary conditions (Eq. (9)). (**b**) Fit of Eq. (9) to points taken from the Supplementary Movie of Ref.^18^ at $t = 10.45$ h. The fitting parameter obtained was ${\mathcal {A}} = 193.9\,\upmu$ m with $R^2 = 0.98$. (**c**) Fit of the same equation to points taken from the Supplementary Movie of Ref.^18^ at $t = 13.95$ h. The fitting parameter obtained was ${\mathcal {A}} = 96.71\mu m$ with $R^2 = 0.938$.

**Figure 6:**
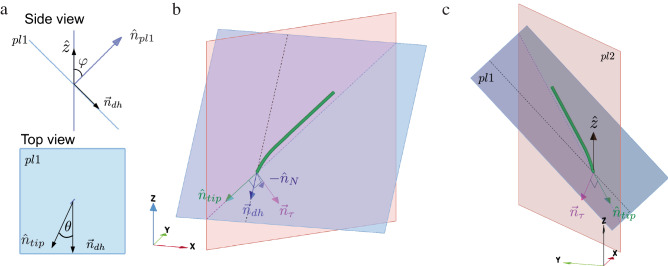
Symmetry breaking: (**a**) Top: a side-view of the plane representing the substrate plane and its normal vector ${\hat{n}}_{pl1}$ (in blue). Bottom: top view of *pl*1 showing the angle $\theta$ between ${\hat{n}}_{tip}$ and $\vec {n}_{dh}$. (**b**) The FMZ (green cylindrical object) and the vectors representing the tip direction $\hat{n}_{tip}$, the downhill direction $\hat{n}_{dh}$, the negative to the normal direction $-{\hat{n}}_N$, and the direction at which force is being applied to the gel $\vec {n}_{\tau }$. The dashed line represents the downhill direction. The bending of the green cylinder represents the bending of the FMZ inside *pl*2. (**c**) Same as (**b**) but from a different viewing angle. Note that the coordinate axes rotate with the different perspectives and are different from the one used in Fig. 3.

To check the hypothesis that waving is a result of buckling, we compare the elastic solution to the dynamical experiments of Thompson and Holbrook^18^ who studied Arabidopsis roots growing on an agar plate tilted at 45$^\circ$. For small deflections, the shape of the neutral line (line inside the rod that is not compressed or stretched) of the buckled rod can be described using the function:

9 $$\begin{aligned} y(x) = {\mathcal {A}}\left( \sin \left( \frac{\beta x}{L}\right) -\frac{\beta x}{L}\cos (\beta )\right) , \end{aligned}$$

obtained by linearizing the equations describing buckling in elastic rods^22^ (this is explained in^24^ and reviewed in the Supplementary Material). Here ${\mathcal {A}}$ is a free parameter not given by the linearized solution (see Fig. 5). In Fig. 5a we show the geometry of the part of the root that is described by Eq. ((9)). In Fig. 5b,c we show fits of Eq. ((9)) to points taken from the neutral line of the highlighted region in Fig. 5a, in two different snapshots of Supplementary Movie 1 in Thompson and Holbrook^18^. We can see that we get a very good fit ($R^2 > 0.93$) by matching the pinned end to the root tip, and the clamped end to the second point of maximal curvature (which is very close to the point where root hairs start to grow) away from the tip (this also sets *L*), and use ${\mathcal {A}}$ as a fitting parameter. In (**c**) we obtained ${\mathcal {A}} = 62.7\,\upmu$ m and in (**d**) ${\mathcal {A}} = 193.9\,\mu$ m. This indicates that the equations that describe the shape of a buckled elastic rod with the pinned-clamped boundary conditions, can describe the shape of the freely moving part of the root during bending, which supports our mechanical explanation for the bending. An important “side effect” of buckling under pinned-clamped boundary conditions, is a change in the direction of the tip. From the experiments we know that in a root that is grown on a horizontal plate, the change in the direction of the tip is monotonic, while for a root that is grown on a tilted plate, the change is oscillating. We claim that this difference is due to a symmetry breaking that stems from the existence of a downhill growth component in the latter case, as will be explained in detail in the next section.

### Symmetry breaking and circular growth

As mentioned above, when grown on flat agar plates, Arabidopsis roots form circular trajectories rather than the usual waving patterns. This leads us to suggest that coiling is the basal growth mode while waving stems from symmetry breaking induced by gravity. However, gravity itself is very weak compared to the other forces such as friction and elasticity and cannot cause the symmetry breaking directly in this case. The symmetry breaking must therefore be a result of growth, which we know is affected by gravitropism. The elongation of the FMZ induces a force that applies in the direction at which the root tip is pointing, and which does not provide any preference to the downhill direction. However, we know that gravitropism causes the root to reorient towards the earth due to differential growth^25–28^ where the cells at the sides of the root farthest from the gel (which is part of the FMZ) grow at a higher rate than the cells closer to the gel. We argue that this effect acted together with root elongation leads to a symmetry breaking. We define ${\hat{n}}_{pl1}$ to be the unit vector normal to the agar surface, which makes an angle $\varphi$ with the vertical axis ${\hat{z}}$ and define ${\hat{n}}_{dh}$ to be the vector pointing in the ‘downhill’ direction:

10 $$\begin{aligned} {\hat{n}}_{dh} = (0,-\cos \varphi ,-\sin \varphi )\,, \end{aligned}$$

as shown in Fig. 6a. We denote the projection of the direction at which the root tip is pointing on the agar plane by ${\hat{n}}_{tip}$ and the angle that it makes with ${\hat{n}}_{dh}$ by $\theta$ (Fig. 6a). Thus, ${\hat{n}}_{tip}$ can be expressed in the component form:

11 $$\begin{aligned} {\hat{n}}_{tip} = (\sin \theta ,-\cos \theta \cos \varphi ,-\cos \theta \sin \varphi ), \end{aligned}$$

as shown in the Supplementary Material (the origin was chosen to be the instantaneous position of the root tip). The bending of the root tip due to gravitropism occurs in a vertical plane *pl*2 that includes ${\hat{n}}_{tip}$ and ${\hat{z}}$, represented by the vector normal to its surface:

12 $$\begin{aligned} {\vec{n}}_{pl2}={\hat{z}}\times {\hat{n}}_{tip}. \end{aligned}$$

Note that, ${\hat{n}}_{tip}$ lies at the intersection of the planes ${\hat{n}}_{pl1}$ and ${\vec{n}}_{pl2}$, as shown in Fig. 6b,c. The bending induced by gravitropism (illustrated by the bending of the green cylinder in Fig. 6b,c) causes the tip to press into the agar and apply a force into the plane of the agar. The direction of the force applied by the root tip on the plane *pl*1, is a vector $\vec {n}_{\tau }$ that lies inside the plane *pl*2 and is perpendicular to the tip direction. It is thus given by (see Supplementary Material for explicit form):

13 $$\begin{aligned} \vec {n}_{\tau } = {\hat{n}}_{tip}\times {\vec{n}}_{pl2}. \end{aligned}$$

The vector $\vec {n}_{\tau }$ has a component going in the direction parallel to the normal to the plane (the direction of $-{\hat{n}}_N=-{\hat{n}}_{pl1}$), a component in the downhill direction ${\hat{n}}_{dh}$, and a component in the transverse direction ${{\hat{x}}}$. When the plate is inclined at $\varphi >0$, the non-vanishing downhill component is:

14 $$\begin{aligned} \vec {n}_{\tau }\cdot {\hat{n}}_{dh} = \sin ^2\theta \sin \varphi , \end{aligned}$$

and when $\varphi <90^\circ$ the non-vanishing normal component is:

15 $$\begin{aligned} \vec {n}_{\tau }\cdot (-{\hat{n}}_{N}) = \cos \varphi . \end{aligned}$$

Finally the transverse component, along the ${{\hat{x}}}$ direction is:

16 $$\begin{aligned} {\hat{n}}_{\tau }\cdot {{\hat{x}}} = -\cos \theta \sin \theta \sin \varphi . \end{aligned}$$

This transverse component causes a retrieving force that counter-balances the change in ${{\hat{n}}}_{tip}$ due to buckling, and tends to orient the tip back to the downhill direction and thus also contributes to the symmetry breaking.

The downhill component breaks the symmetry since it causes any slip event, that occurs when the force applied by the root tip due to growth becomes larger than the maximal static friction, to have a downhill component for a finite $\varphi$. For that reason, whenever the root tip overcomes the static friction, it has a component in the downhill direction, guaranteeing that the average growth direction will be downhill, which is consistent with waving but not with coiling. However, when the root is grown on a flat plate ($\varphi =0^\circ$), the downhill force component vanishes and the tip direction keeps changing in the same direction.

## Experimental results

**Figure 7:**
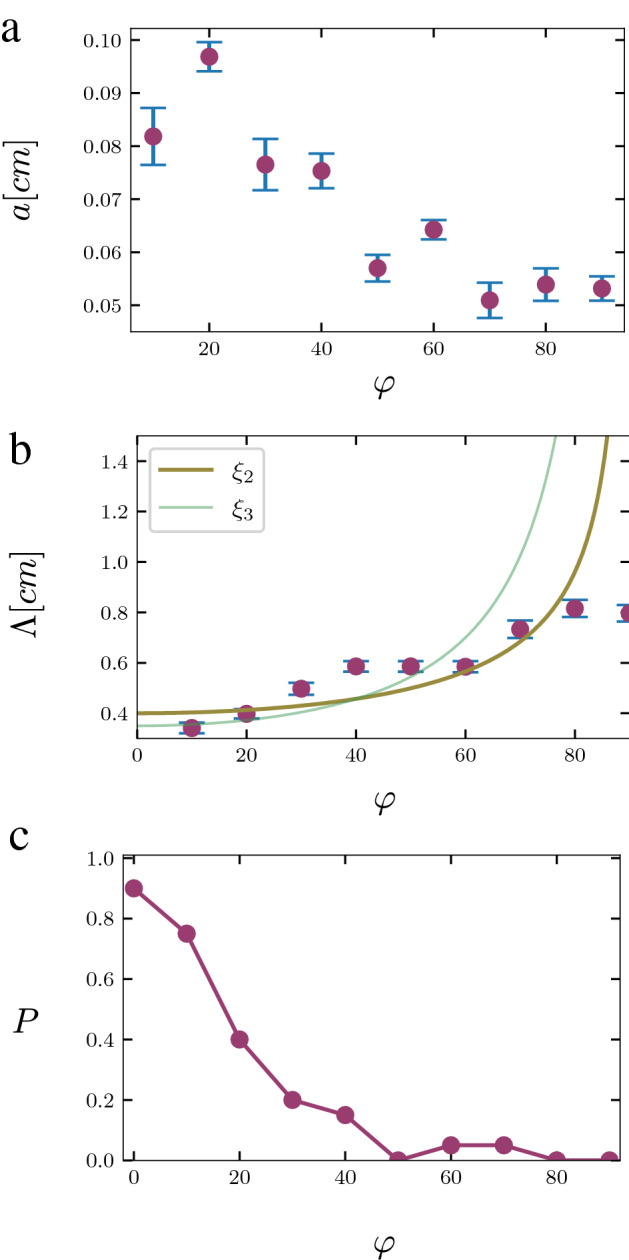
Experimental results: (**a**) Change in amplitude as a function of tilt angle. (**b**) Change in wavelength as a function of tilt angle compared to Eq. (18) (thick dark-yellow line) and Eq. ((19)) (thin green line). (**c**) Probability of a root to form coils as a function of the angle. In all cases the sample size was $n=20$ and the standard error is represented by vertical lines.

To further explore the role played by mechanics in forming waving and coiling patterns, we studied the effect of changing the tilt angle by growing *Arabidopsis thaliana* L. cv. Columbia seedlings on agar hydrogel surfaces at different angles $\varphi = 0^\circ , 10^\circ , 20^\circ , 30^\circ , 40^\circ , 50^\circ , 60^\circ , 70^\circ , 80^\circ , 90^\circ$ with respect to the vertical. To control and change the tilt angle, we used growing platforms that were 3D printed at the different tilt angles (see Fig. 1d and “Materials and methods” section for details). To prevent phototropic effects, we used printed barriers to separate the seedlings roots from the shoots, where the latter were exposed to light and the former were covered. A microscope was used to analyze each sample after the roots started to approach the edges of the plate, and for each root it was determined whether it exhibits waving or coiling. For the roots that showed waving, the amplitude and wavelength were recorded (Fig. 7a,b).

We first studied the effect of the tilt angle on coiling. We have found that there is a gradual transition from waving to coiling (Fig. 7c). This is contrary to our expectation of a sharp transition from waving to coiling at $\varphi = 0^\circ$ (i.e. when there is no downhill direction). We believe that this is a result of the fact that the surface of the agar is not completely flat, which could cause the root to become blocked on a crease on the surface. This assumption is supported by the fact that in numerous cases, coiling occurred after a long period of waving (an example is shown in Fig. 1c), when the agar was already relatively dry and that it is known that drying causes gels to deform elastically and form periodic patterns^29^.

**Figure 8:**
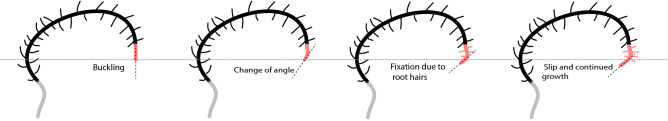
A growth and buckling step giving rise to a circular growth pattern. Each step starts with a buckling event, that causes a small change of angle. This small change of angle is fixed to the gel by the advancing root hairs that causes the free zone to become shorter and “straighter”. However, the FMZ does not become completely straight and when it grows and buckles again, it tends to bend in the same direction in the absence of the symmetry breaking component of the force applied by the root tip.

In the cases where waving was observed, both wavelength and amplitude were affected by the tilt angle. The wavelength showed a strong increase while the amplitude showed a moderate decrease with the tilt angle. We have also found that the growth dynamics and patterns differed from the observations of Thompson and Holbrook^18^. In the movies by Thompson and Holbrook, it is clear that the wavelength is comparable to the size of the FMZ, while in our experiments the wavelength was much larger than the length of the FMZ (see Movie 1 in Supplementary Material). We would like to stress that our results are consistent with other experimental results^14^ which suggests that the wavelength is very sensitive to environmental factors (specifics of lighting, temperature, agar preparation etc.).

We did find an example where buckling was clearly visible in our experiments, but only under very specific conditions where the agar was very dry. In this case, shown in Supplementary Movie 3, we observed buckling without slip. We fitted Eq. (9) to two consecutive snapshots and found that the snapshot just after buckling agreed with the linearized solution, while the next snapshot did not. We believe that the reason is that since the root tip did not progress, the curvature reached the nonlinear regime and the linearized solution was not suitable anymore (Supplementary Fig. 2). In Supplementary Movies 1 and 2 we were not able to check for buckling behavior since the time scale of the “bending” phase in the stick-grow-bend-slip dynamics is short compared to the time between taking images and because the images were not sharp enough to allow the observation of the root hairs.

Nevertheless, Supplementary Movie 3 does indicate that friction and elasticity play a role in our experiments and for that reason and because of the fact that in both our experiments and the experiments of Thompson and Holbrook^18^, waving patterns were observed, we believe that the dynamics observed in our experiments can be explained using the same mechano-sensing considerations that were discussed above. As can be seen in the movies by Thompson and Holbrook^18^, the increase in curvature is gradual and between different episodes of curving, there are short bursts of slip. In our experiments (Movie 1 in Supplementary Material), the change in direction is very gradual and is accompanied by a seemingly continuous slip. Similar behavior is observed in circular growth (Movie 2 in Supplementary Material). Here we suggest that all cases stem from the same mechanism, illustrated in Fig. 8. The idea is that similarly to stick-grow-slip in a straight line (see Supplementary Material), when the FMZ slips after a period of curving, it becomes arrested in a configuration that is not completely stress-free. Since in this case, the accumulation of elastic energy due to growth is caused by bending, when the tip becomes arrested, the FMZ still has some curvature. This causes the new “stuck” configuration to have some initial curvature, and as we have shown, this means that the curvature will tend to increase further until it becomes suppressed by the growth of root hairs and/or by another slip event. In the case of waving, the part of the force applied by the root tip that is pointing in the downhill direction, causes the tip to change direction eventually and thus gives rise to a waving pattern. However, when the plate is horizontal ($\varphi =0^\circ$), this component is absent and thus the root tip keeps rotating in the same direction, causing the growth to become circular.

To demonstrate the relevance of the mechanical considerations to our experimental observations, we relate the wavelength to the tilt angle using dimensional analysis. We hypothesise that the wavelength $\Lambda$ is a length-scale that arises from the relevant dimensional quantities in the problem: the average force applied by the root tip into the plate which is proportional to $\vec {n}_{\tau }\cdot {\hat{n}}_N$ and thus, according to Eq. (15), can be written as $F_0\cos \varphi$ ($F_0$ is a constant with dimensions of force), the bending modulus *B*, the average length of the freely-moving zone $\langle L\rangle$ and the growth rate *k*. From these quantities we obtain the length-scales:

17 $$\begin{aligned} \xi _1= & {} \langle L\rangle \end{aligned}$$

18 $$\begin{aligned} \xi _2 \sim \sqrt{\frac{B}{F}}= & {} \sqrt{\frac{B}{F_0\cos \varphi }}, \end{aligned}$$

and:

19 $$\begin{aligned} \xi _3 \sim \frac{B}{\langle L\rangle F} = \frac{B}{\langle L\rangle F_0\cos \varphi }. \end{aligned}$$

Since the wavelength changes with the angle $\varphi$, we assume that either $\Lambda = \xi _2$ or $\Lambda = \xi _3$ (note that *k* does not affect these length-scales). In Fig. 7b we can see that $\xi _2$ agrees somewhat better with the experimental results though the difference is small. The last two expressions show the same general trends as the experimental results for the wavelength: when $\varphi = 0^\circ$ (petri-dish is flat) the wavelength is minimal, while when $\varphi = 90^\circ$ (petri-dish is vertical), the wavelength is infinite, which would correspond to non-waving. However, in the experiments there is always a finite wavelength, even when the plate is completely vertical. At this angle the waving pattern also shows some randomness. We believe that the randomness and the fact that the wavelength is finite when $\varphi = 90^\circ$, are both a result of the fact that the agar surface is never completely flat, which is probably also the reason that the theoretical curve does not look exactly the same as the experimental results. However, for $\varphi \le 80^{\circ }$, $\xi _2$ agrees with the trend observed in experiments (Fig. 7b).

As a further check of the role of mechanics in waving, we have repeated the experiments with several plant species and found a correlation between the thickness of the root and the occurrence of waving and coiling. We have measured the root thickness of several species and found it to be: $r = 0.062 \pm 0.004\, \text {mm}$ in Arabidobsis, $r = 0.068 \pm 0.005\, \text {mm}$ in Eutrema, $r = 0.123 \pm 0.01\, \text {mm}$ in Tobacco and $r=0.260 \pm 0.005\, \text {mm}$ in Tomato. We have found that thicker roots tended to show less waving and coiling. The waving and coiling of Eutrema were similar to Arabidobsis, while the thicker Tobacco showed waving and coiling only at rather shallow angles (close to $\varphi =90^\circ$). Tomato, the plant with the thickest roots, did not show waving and coiling at all. These results support the notion that waving is a result of buckling since the bending modulus *B*, and thus also the buckling force $F_B$, strongly depend on the root radius *r* ($B\sim r^4$).

## Discussion

In this work we studied root waving and coiling theoretically and experimentally. We showed, theoretically, that important aspects of the phenomenology observed can be explained by studying the dynamics of a hairless region (the freely moving zone—FMZ) close to the root tip, and taking into account growth, gravitropism and mechanics. The main hypothesis is that waving results from a combination of stick-grow-slip dynamics with elastic buckling of the FMZ, that is induced by growth limited by friction. This hypothesis is used to perform calculations that are then compared to experimental results obtained in previous work as well as to new experimental results that we obtained. We show that the theoretical results can explain many of the features of the observed phenomena, including the dependence of wavelength on plate tilt angle, the shape of the root during different stages of waving and the fact that thick roots do not show waving.

Our experiments reveal that growth dynamics is very sensitive to environmental factors—in the experiments performed by Thompson and Holbrook, the wavelength was comparable to the size of the FMZ and buckling and intermittent slipping of the root tip were clearly observed, whereas in our experiments the wavelength was much larger than the FMZ, and the observed slip and change in growth direction were seemingly smooth. However, we suggest that the same basic mechanism occurs in all cases and explain qualitatively how the different behaviors can be reconciled.

There are still many open questions that were left for future work. First, static friction and stick-grow-slip dynamics is not observed directly and observing it will require new experiments. Second, the theoretical calculations do not agree with the experimental observations in the limit of vertical slopes and the transition from waving to coiling is not fully explained using our current modeling approach, that does not take into account noise. Furthermore, the current calculations do not take into account root hair growth explicitly, and do not form a full model of the dynamics. Also, we do not explain the periodic twisting of the FMZ that was observed experimentally^12,18^. We believe that the rotation can be explained as rolling without slipping of the root tip, that allows for bending of the FMZ without friction, but a more exact theoretical and experimental analysis of the effect will be left for future work (see Fig. 3). Finally, while we believe that our experimental and theoretical results do point to the scenario in which the response of roots to obstacles is a result of the combined effect of mechanical constraints and growth rather than the ability to sense forces, further study will be required to verify this hypothesis.

## Materials and methods

The experimental protocol comprised of two stages—in the first stage, sterilised seeds were germinated on a 0.8% soft MS (Murashige and Skoog^30^) agar culture. agar hydrogels were mixed with 0.5 g/L MES buffer, 15 g/L Sucrose and a KOH solution that was used to set the pH to 5.7^31^. The gels were then autoclaved for 1 h before they were poured into a circular Greiner^®^  petri dish. The agar thickness was controlled such that it had an average depth of 1 cm which limited dehydration. For Eutrema, which is sucrose stress sensitive^32,33^, we used a sucrose-less agar (*Eu* agar). After the agar was poured into the plates and the seedlings were added, the plates were sealed using a 3 M$^{\circledR }$ porous surgical tape for infection prevention. In the seedlings preparation stage, the seeds were surface-sterilized in 50% NaOCl bleach for 10 min, and were washed three times using sterile water. At this stage the agar plates were placed at 4 °C environment for 4 to 5 days and then moved to a growth room. The seeds were next germinated in a growth room kept at a temperature of 24 °C and the agar plates were exposed to white fluorescent light bands (light intensity: approximately 180 $\upmu \text {mol}$ m^−2^ s^−1^) 16 h light, 8 hours dark. Once the seeds germinated and the plants lengths reached approximately 100 mm, the seedlings were taken from the soft agar and placed on square plates with 1% agar density on which root growth and imaging took place.

**Figure 9:**
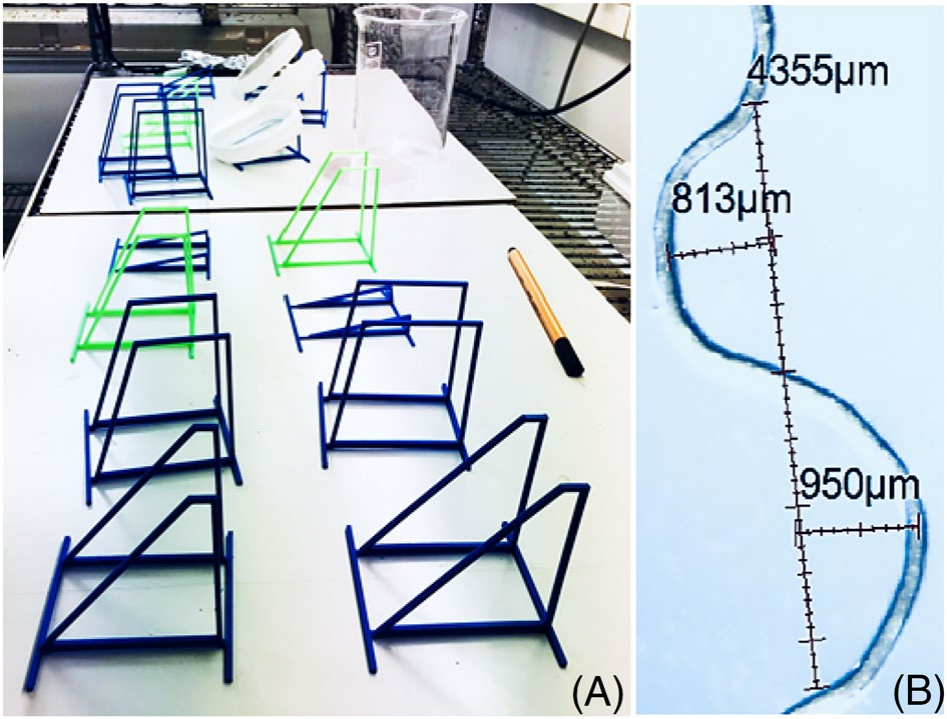
Experimental setup: (**A**) Growing platforms 3D printed in different inclination angles $\varphi$ that were used to place plates containing agar surfaces on which the roots were grown. (**B**) An example of wavelength and amplitude measurements.

These petri dishes were placed on 3D printed supporters that were prepared such that the plate was tilted in angles varying between 10 $^\circ$ and 90$^\circ$ (see Fig. 9A). To avoid phototropic effects (growing in the opposite direction of a light source^12,34,35^) the plants were cultured under fluorescent light bands (light intensity: approximately 270 $\upmu \text {mol}$ m^−2^ s^−1^) and the part of the petri dish containing the roots was covered by an aluminum paper. All the samples were grown in a plant growing room, and all the experiments and measurements were performed at room temperature of 24$^\circ$C (± 2$^\circ$ C).

A digital photography analysis system composed of a Nikon Stereo-zoom microscope (Nikon SMZ 745 T), DeltaPix$^{\circledR }$ microscope camera (Invenio 5SIII—Microscope camera with 5 Megapixel resolution and CMOS sensor) and a computer installed DeltaPix InSight V5.3.6$^{\circledR }$ and Adobe Photoshop$^{\circledR }$, were used for measuring the root wavelength $\Lambda$ and root wave amplitude *a* after the end of a growing period. From the images (Fig. 9B, for example) we estimate an approximate neutral line about which the root oscillates, and calculate the average amplitude and wavelength using that as a reference line. We therefore assume an error of the order of the radius *r*, which for the wavelength $\Lambda$ comes out to be $\sim 0.4{-}6\%$ and for the amplitude it is 6–10%. The relative error is largest for small amplitudes and wavelength and becomes smaller as the amplitude and wavelength increases. For most wavelength values the measurement error is significantly smaller than the standard deviation.

### Plant material declaration

Experimental research involving plant material complied with relevant institutional, national and international guidelines and legislations. Seed collection was performed according to the relevant permissions.

## Supplementary Information

Supplementary Information.

Supplementary Movie 1.

Supplementary Movie 2.

Supplementary Movie 3.
