# Supplementary material for: A mechano-sensing mechanism for waving in plant roots
Source: Sci Rep. 2022 Jun 10;12:9635. doi: 10.1038/s41598-022-14093-1 (PMC9187721; doi:10.1038/s41598-022-14093-1)
Supplement: Supplementary file 1 — Supplementary Information. [file 41598_2022_14093_MOESM1_ESM.pdf]

Supplementary material for: A Mechano-Sensing Mechanism for Waving in Plant Roots

Growth of a one dimensional rod

As stated in the main text, we treat the freely moving zone as a growing rod clamped on one side by the root hairs and pinned on the other side by friction. Following Goriely [1], when describing a growing rod, there are three different configurations:

- The initial configuration $\mathcal{B}_0$ - the configuration before growth has started. Positions inside this configuration are described using the arc-length, denoted by S_0, which varies between 0 and L_0.
- The "virtual" configuration $\mathcal{V}(t)$, where t is the time. This is an unstressed deformation of $\mathcal{B}_0$ due to growth ($\mathcal{V}(0) = \mathcal{B}_0$). The arclength of this configuration is denoted S and its length is L (when there is growth $L(t) > L_0$ for $t > 0$). It is called a "virtual" configuration since the boundary conditions force the rod to deform to a different configuration.
- The current configuration $\mathcal{B}(t)$ is the actual (stressed) configuration at time t. It is an elastically deformed, virtual configuration $\mathcal{V}$, that is subject to the actual boundary conditions. The arclength of this configuration is denoted S and its length is ℓ.

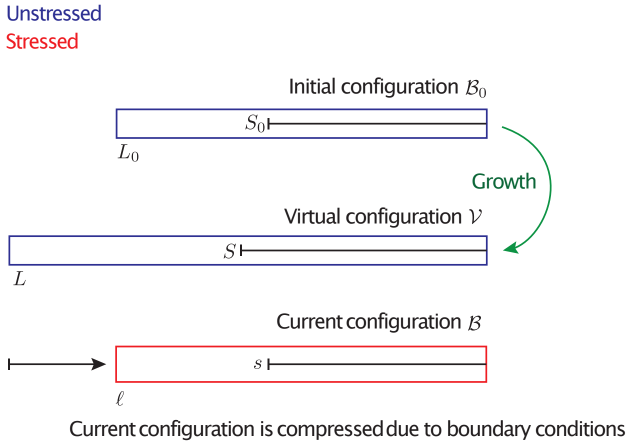

FIG. 1. The different coordinate systems used to describe growth in an elastic rod.

If the rod is not subject to any external forces or confinement, its differential deformation will be:

$$\gamma = \frac{\partial S}{\partial S_0} \tag{1}$$

where $S(t)$ changes with time. If the ends of the rod are constrained to stay at fixed positions, or it is subject to body forces, its shape may change elastically and the amount of deformation will be:

$$\alpha = \frac{\partial s}{\partial S} \tag{2}$$

When both growth and elasticity play a role, the total stretch with respect to the initial configuration will be:

$$\lambda = \frac{\partial s}{\partial S_0} = \frac{\partial s}{\partial S}\frac{\partial S}{\partial S_0} = \alpha\gamma \tag{3}$$

If we assume linear elasticity (this should be the case for a relatively unstretchable material such as a plant root), the stress will be:

$$\sigma_{xx} = E(\alpha - 1) = E\epsilon \tag{4}$$

where ϵ is the strain. Here and in the following, we assume that the growth is much slower than any elastic or dissipative response (the growth rate is orders of magnitude slower than the speed of sound) and therefore, we can

assume that the material is in mechanical equilibrium at every given moment. The deformation rate due to growth will be governed by some growth rule:

$$\frac{\partial\gamma}{\partial t} = G(\gamma, s, S_0) \tag{5}$$

and can depend on the microscopic mechanism which allow for growth in a specific organ.

Stress accumulation due to growth

If the rod is held at a constant length but is allowed to grow, γ will increase but λ will be constant ($\lambda = 1$). Here we assume uniform growth at the FMZ:

$$\frac{\partial\gamma}{\partial t} = \begin{cases} k & 0 < S_0 < L_0 \\ 0 & S_0 > L_0 \end{cases} \tag{6}$$

For a uniform growth:

$$\gamma = 1 + kt \tag{7}$$

(assuming $\gamma(0) = 1$) and:

$$\alpha = \frac{\lambda}{\gamma} = \frac{\lambda}{1+kt} = \frac{1}{1+kt} \tag{8}$$

the stress will become nonzero and will become more negative over time due to confinement (the rod becomes compressed):

$$\sigma = E(\alpha - 1) = E\left(\frac{1}{1+kt} - 1\right) \tag{9}$$

Stick-slip without buckling

- **Stick phase** (λ constant, γ increasing):

$$\sigma_{xx} = E\left(\frac{1}{1+kt} - 1\right) = -E\left(\frac{kt}{1+kt}\right) \tag{10}$$

the stress is a result of the static friction applied on the root tip on one side of the FMZ and the clamping by the root hair on the other side of the FMZ, both resisting the expansion. The force that the boundaries apply on the rod is:

$$F_x = \sigma_{xx} A = -AE\left(\frac{kt}{1+kt}\right) \tag{11}$$

this is equal in magnitude but opposite in direction to the friction force:

$$f = \begin{cases} AE\left(\frac{kt}{1+kt}\right), & \text{if } |f| \le \mu_s F_N \\ \mu_k F_N, & \text{otherwise} \end{cases} \tag{12}$$

when $F_x = \mu_s F_N$ the tip slides until it becomes arrested again due to the kinetic friction $f_k = \mu_k F_N$. The waiting time before the onset of a slip event can be found from the equation:

$$AE\left(\frac{kt_{slip}}{1+kt_{slip}}\right) = \mu_s F_N \tag{13}$$

$$t_{slip} = \frac{\mu_s F_N}{k(AE - \mu_s F_N)} \tag{14}$$

this also defines the stretch at slipping γ_{slip}:

$$AE\left(1-\frac{1}{\gamma_{slip}}\right)=\mu_s F_N \tag{15}$$

$$\gamma_{slip}=\frac{AE}{AE-\mu_s F_N} \tag{16}$$

after the onset of a slip event, the tip moves until kinetic friction causes the tip to become arrested in a less stressed position.

- **Slip phase** (λ increasing, γ constant): We assume that the growth is too slow to increase significantly during a slip event and therefore we assume that γ is constant and ℓ is increasing as:

$$\ell=L_0+\int_{t_{slip}}^{t} v(t')dt' \tag{17}$$

where $v(t)$ is the tip velocity. The elongation is thus:

$$\lambda=\frac{\ell}{L_0}=1+\frac{1}{L_0}\int_{t_{slip}}^{t} v(t')dt'=1+\frac{1}{L_0}x(t) \tag{18}$$

$$x(t)=L_0(\lambda-1) \tag{19}$$

where $x(t)$ is the position of the root tip with respect to its position at $t=0$. From this we also get that:

$$\dot{x}(t)=L_0\frac{d\lambda}{dt} \tag{20}$$

and:

$$\ddot{x}(t)=L_0\frac{d^2\lambda}{dt^2} \tag{21}$$

The elastic force is:

$$F_{el}=-F_x=-\sigma_{xx}A=AE(\alpha-1)=AE\left(1-\frac{\lambda}{\gamma_{slip}}\right) \tag{22}$$

since $\gamma_{slip}>\lambda$ this force is initially positive $F_{el}(0)>0$. The equation of motion is:

$$m\ddot{x}=F_{el}-f_k=-AE\left(\frac{\lambda}{\gamma_{slip}}-1\right)-f_k \tag{23}$$

where m is an effective mass representing the inertia of the root tip which we approximate as a point particle. The root tip will become arrested after F_{el} becomes smaller than f_k. Substituting:

$$\frac{d^2\lambda}{dt^2}=-\frac{AEL_0}{m}\left(\frac{\lambda}{\gamma_{slip}}-1\right)-\frac{L_0}{m}\mu_k F_N \tag{24}$$

$$\frac{d^2\lambda}{dt^2}=-\frac{AEL_0}{m\gamma_{slip}}\left(\lambda-\gamma_{slip}+\frac{\gamma_{slip}}{AE}\mu_k F_N\right) \tag{25}$$

the equation of motion is then:

$$\frac{d^2\lambda}{dt^2}=-\omega^2\left(\lambda-\lambda_0\right) \tag{26}$$

where:

$$\omega = \sqrt{\frac{AEL_0}{m\gamma_{slip}}} \tag{27}$$

$$\lambda_0 = \gamma_{slip}\left(1 - \frac{\mu_k F_N}{AE}\right) \tag{28}$$

define $\epsilon = \lambda(t) - \lambda_0$:

$$\ddot{\epsilon} = -\omega^2 \epsilon \tag{29}$$

$$\epsilon = A\cos(\omega t + \phi) \tag{30}$$

$$\lambda(t) = A\cos(\omega t + \phi) + \lambda_0 \tag{31}$$

the initial conditions are:

$$\lambda(t_{slip}) = A\cos(\omega t_{slip} + \phi) + \lambda_0 = 1 \tag{32}$$

$$\dot{\lambda}(t_{slip}) = -A\omega\sin(\omega t_{slip} + \phi) = 0 \tag{33}$$

from which we get $\phi = -\omega t_{slip}$ and $A = 1 - \lambda_0$:

$$\lambda(t) = (1 - \lambda_0)\left(\cos(\omega(t - t_{slip})) - 1\right. \tag{34}$$

the root tip stops moving when $\dot{\lambda} = \dot{x} = 0$:

$$\dot{\lambda} = -\omega\lambda_0 \sin(\omega(t - t_{slip})) = 0 \tag{35}$$

which occurs when:

$$\Delta t = t_{stop} - t_{slip} = \frac{\pi}{\omega} \tag{36}$$

$$t_{stop} = \frac{\pi}{\omega} + t_{slip} = \pi\sqrt{\frac{m\gamma_{slip}}{AEL_0}} + \frac{\mu_s F_N}{k(AE - \mu_s F_N)} \tag{37}$$

the deformation after the tip has stopped will be:

$$\lambda(t_{stop}) = A\cos(\omega(t_{stop} - t_{slip})) + \lambda_0 = A\cos(\pi) + \lambda_0 = -A + \lambda_0 = 2\lambda_0 - 1 \tag{38}$$

the displacement after the tip has stopped will be:

$$x_{stop} = L_0(\lambda - 1) = L_0(2\lambda_0 - 2) = 2L_0(\gamma_{slip}\left(1 - \frac{\mu_k F_N}{AE}\right) - 1) \tag{39}$$

Estimation of length- and time-scales for stick-slip

We obtain a rough estimate of the length- and time-scales involved in the stick-slip dynamics from equations 27,39. For Arabidopsis:

$$A = \pi r^2 = 1.2 \times 10^{-8} m^2 \tag{40}$$

We do not have values of Young's modulus for Aabidopsis. We therefore use the measurements of the Young's modulus of Medicago obtained in [2]:

$$E \approx 5.5 \times 10^7 Pa \tag{41}$$

and assume that it is of the same order of magnitude as Young's modulus in Arabidopsis. For the force we use a typical value obtained from experiments on different plants [3]:

$$F_N = 6 \times 10^{-3} N \tag{42}$$

the length of the FMZ is approximately:

$$L_0 = 10^{-3} m \tag{43}$$

For small normal forces, the friction coefficients of gels are found to be similar to those of solid friction [4]. We choose:

$$\mu_s = 0.3 \tag{44}$$

$$\mu_k = 0.2 \tag{45}$$

Substituting in the expression for the slip length, we obtain:

$$\gamma_{slip} = \frac{AE}{AE - \mu_s F_N} \sim 1.0027 \tag{46}$$

and:

$$x_{stop} = L_0(\lambda - 1) = L_0(2\lambda_0 - 2) = 2L_0(\gamma_{slip}\left(1 - \frac{\mu_k F_N}{AE}\right) - 1) \sim 2\mu m\,. \tag{47}$$

For the slip time we need to calculate the frequency:

$$\omega = \sqrt{\frac{AEL_0}{m\gamma_{slip}}}\,. \tag{48}$$

We therefore need to estimate the mass of the FMZ. We estimate it to be 1 mg $= 10^{-6}$ kg. The slip time is thus:

$$\Delta t = \frac{\pi}{\omega} \sim 0.2s\,. \tag{49}$$

The time and length scales are small, as expected, but the motion should be possible to detect in a microscope with a fast camera.

Rods in two dimensions and buckling

When the rod is allowed to grow on a plane (this is known as Euler's elastica), we must balance both forces $\vec{F}(s)$ and torques $\vec{M}$. This leads to the following classical equations of the elasticity of thin rods [5]:

$$\frac{d\vec{F}}{ds} = 0 \tag{50}$$

$$\frac{d\vec{M}}{ds} - \vec{F} \times \hat{t} = 0 \tag{51}$$

where

$$\hat{t} = \left(\frac{dx}{ds}, \frac{dy}{ds}\right) = (\cos\theta(s), \sin\theta(s)) \tag{52}$$

is a unit vector tangential to the rod and $\theta(s)$ is the angle that $\vec{t}$ forms with the x-axis. The coordinates $(x(s), y(s)$ are the coordinates of the "neutral line" - the line on which an inextensible rod does, the material is not stretched or compressed (see Fig 3 in main text). Writing the formulas in a more explicit form and using the constitutive relation for thin elastic rods, connecting the moment to the curvature:

$$M = B\kappa = B\frac{d\theta}{ds}\,. \tag{53}$$

We get that when in mechanical equilibrium, the elastic rod (our FMZ) must satisfy the equations [6]:

$$\frac{dF_x}{ds} = 0\,; \qquad \frac{dF_y}{ds} = 0 \tag{54}$$

$$B\frac{d^2\theta}{ds^2} + F_x \sin\theta - F_y \cos\theta = 0\,. \tag{55}$$

For a pinned FMZ, the boundary conditions are:

$$y(0) = 0\,, M(0) = B\frac{d\theta}{ds}(0) = 0\,; \qquad y(L) = 0\,, \theta(L) = 0 \tag{56}$$

since it is pinned by the friction on the other end (the root tip), and clamped on the other end by the root hairs (it cannot change the angle at all there). To obtain a dimensionless form, we rescale the equations by $s = L\tilde{s}$ and use the fact that initially $F_y = 0$ (assume that the rod is oriented in the x direction):

$$\frac{d^2\theta}{d\tilde{s}^2} + \frac{F_x L^2}{B}\sin\theta = 0 \tag{57}$$

For small angles we can linearise the equation by assuming that:

$$x \approx s, \qquad \theta = \frac{dy}{dx} \tag{58}$$

Substituting, and assuming that $\sin\theta \approx \theta$, we obtain the equation:

$$B\frac{d^3y}{dx^3} + F_x\frac{dy}{dx} = 0 \tag{59}$$

we take the derivative with respect to x to take into account the equilibrium condition $\frac{dF_x}{dx} = 0$ [7]:

$$B\frac{d^4y}{dx^4} + F_x\frac{d^2y}{dx^2} = 0 \tag{60}$$

the boundary conditions are now:

$$y(0) = 0,\ \frac{d^2y}{dx^2}(L) = 0, \qquad y(L) = 0,\ \frac{dy}{dx}(L) = 0 \tag{61}$$

this equation has a straight solution $y(x) = 0$ which stable for small forces. However, for F_x larger than a critical value:

$$F_B = \frac{\beta^2 B}{L^2} \tag{62}$$

The buckled solution:

$$y(x) = A\left(\sin\left(\frac{\beta x}{L}\right) - \frac{\beta x}{L}\cos(\beta)\right) \tag{63}$$

becomes stable and the $y(x) = 0$ solution becomes unstable to perturbations (for more details see [7]). Note that:

$$\beta^2 \approx 20.19073 \tag{64}$$

is found from the boundary condition $y(L) = 0$, which leads to the equation:

$$\tan(\beta) = \beta \tag{65}$$

The time, force and length at which buckling occurs can be found by finding the first time at which the buckling condition is satisfied:

$$L^2 F_x = \beta^2 B \tag{66}$$

substituting:

$$L_0^2(1+kt)^2 AE\left(\frac{kt}{1+kt}\right) = \beta^2 B \tag{67}$$

and solving, we find:

$$t_b = \frac{1}{2k}(\sqrt{1+4c^2} - 1) \tag{68}$$

where:

$$c = \frac{r\beta}{2L_0} \tag{69}$$

Post-buckling in experiments

In the main text we showed that the shape of waving in the movie published by Thompson and Holbrook [8] can be fitted to the buckling equation 63. In our waving and coiling movies it is difficult to observe buckling since the time scale of the "sticking" phase in the stick-grow-bend-slip dynamics is short compared to the time between taking images and because the images were not sharp enough to allow the observation of the root hairs. However, in one case, where the gel was relatively dry, the root tip became stuck in one point on the gel and showed clear buckling. This is shown in Supplementary movie 3. In Fig 2 we show fits to the first two snapshots taken from this movie. We can see that the fit to the first snapshot is rather good with $R^2 = 0.972$ while the fit to the second snapshot is not good with $R^2 = 0.617$. We believe that the reason is that because the root tip could not move, the root reached curvatures that cannot be described by the linearized solution 63.

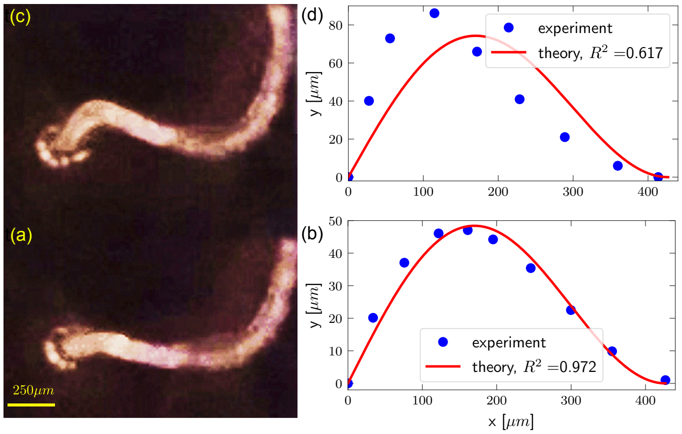

FIG. 2. **Analysis of the buckling observed in supplementary movie 3** (a,c) are images showing the shape of the FMZ at an early time (a) and a later time (c). (b,d) are fits of the theory to representative points taken from the center line of (a) and (c) respectively.

Force in the post-buckling regime

We start by rescaling the equations by $s = L\tilde{s}$:

$$\frac{d^2\theta}{d\tilde{s}^2} + \frac{L^2}{B} F_x \sin\theta - \frac{L^2}{B} F_y \cos\theta = 0 \tag{70}$$

which allows us to write equation 55 in a dimensionless form:

$$\frac{d^2\theta}{d\tilde{s}^2} + \tilde{F}_x \sin\theta - \tilde{F}_y \cos\theta = 0 \tag{71}$$

where:

$$F_x = \frac{B}{L^2}\tilde{F}_x\,, \qquad F_y = \frac{B}{L^2}\tilde{F}_y \tag{72}$$

Following the perturbation theory by Wang (for more details see [6]), we expand $\theta(s)$ for a small parameter ϵ:

$$\theta(s) = \epsilon\theta_0(s) + \epsilon^3\theta_1(s) + O(\epsilon^5) \tag{73}$$

the dimensionless force changes nonlinearly with ϵ:

$$\tilde{F}_x = a_0 + a_1\epsilon^2 + O(\epsilon^4) \tag{74}$$

$$\tilde{F}_y = b_0\epsilon + b_1\epsilon^3 + O(\epsilon^5) \tag{75}$$

where a_0 and a_1 are dimensionless constants. Similarly, the displacement at the tip (i.e its position with respect to its undeformed position) is:

$$u = u_0\epsilon^2 + O(\epsilon^4) \tag{76}$$

where u_0 is a dimensionless constant. We invert the asymptotic series to obtain (for justification see [9]):

$$\epsilon^2 = \frac{u}{u_0} + O\left(u^2\right) \tag{77}$$

The constants a_0, a_1 and u_0 are obtained from the perturbative solution. Substituting:

$$\tilde{F}_x^2 = a_0^2 + 2a_0a_1\epsilon^2 + O(\epsilon^4) \tag{78}$$

$$\tilde{F}_y^2 = b_0^2\epsilon^2 + O(\epsilon^4) \tag{79}$$

$$\tilde{F} = \sqrt{\tilde{F}_x^2 + \tilde{F}_y^2} \approx \sqrt{a_0^2 + (2a_0a_1 + b_0^2)\epsilon^2} \tag{80}$$

expanding:

$$\tilde{F} \approx a_0 + \frac{1}{2a_0}(2a_0a_1 + b_0^2)\epsilon^2 \approx a_0 + \frac{1}{2a_0}(2a_0a_1 + b_0^2)\left(\frac{u}{u_0}\right) \tag{81}$$

We assume that the tip was pinned at buckling and therefore $L(t_b) = L_B$, where t_b is the time at which buckling occurs. The displacement at the tip is therefore:

$$u = \frac{L - x(L_0)}{L} = \frac{L - L_B}{L} \tag{82}$$

for a static configuration, the relation between the force and the rod length L is (substituting the expression for the dimensionless displacement):

$$\tilde{F} \approx a_0 + \frac{1}{2a_0}(2a_0a_1 + b_0^2)\left(\frac{1}{u_0}\left(\frac{L - x(L_0)}{L}\right)\right) = a_0 + \mathcal{C}\left(\frac{L - L_B}{L}\right) \tag{83}$$

where:

$$\mathcal{C} = \frac{2a_0a_1 + b_0^2}{2a_0u_0} \tag{84}$$

In the post-buckling period, the main contribution to the energy comes from bending and, in principle, we can ignore stretching or compression. We can therefore assume approximately that:

$$\lambda = \gamma\,, \qquad L = \ell \tag{85}$$

and thus:

$$\frac{\partial \lambda}{\partial t} = k \tag{86}$$

$$\lambda = 1 + kt \tag{87}$$

and since we are starting from the buckling length L_B and buckling time t_b:

$$L = L_B(1 + k(t - t_b)) \tag{88}$$

substituting, the dimensionless force as a function of time will then be:

$$\tilde{F} \approx a_0 + \mathcal{C}\left(\frac{k(t-t_b)}{1+k(t-t_b)}\right) \tag{89}$$

the dimensional force F will be (note that $a_0 = \beta^2$, see [6]):

$$F \approx \frac{B}{L^2}\left[a_0 + \mathcal{C}\left(\frac{k(t-t_b)}{1+k(t-t_b)}\right)\right] = \frac{B}{L_B^2(1+k(t-t_b))^2}\left[a_0 + \mathcal{C}\left(\frac{k(t-t_b)}{1+k(t-t_b)}\right)\right] \tag{90}$$

$$= F_B\frac{1}{(1+k(t-t_b))^2}\left[1 + \frac{\mathcal{C}}{a_0}\left(\frac{k(t-t_b)}{1+k(t-t_b)}\right)\right] \tag{91}$$

which is a decreasing function of time (see Fig 4 in the main text). This means that growth becomes easier for the plant after bending. An intuitive explanation is that for a longer rod the force required to buckle it is smaller and therefore, to reach a certain post-buckled state will also require less force. Note that the obtained expression for the post-buckling force is of the form:

$$F = F_c(1 + a\delta + b\delta^2 + ...) \tag{92}$$

that is used to describe post-buckling behavior in different mechanical systems[10], Here δ is the deflection, F is the force and F_c is the critical (buckling) force.

FORCE LEADING TO SYMMETRY BREAKING

We describe the substrate plane (the plane on which the root grows - blue plane in Fig 7(a) in the main text) using the equation:

$$\hat{n}_{pl1} \cdot (\vec{r} - \vec{r}_0) = \hat{n}_{pl1} \cdot \vec{r} = 0\,, \tag{93}$$

where we choose the position of the root tip to be the origin $\vec{r}_0 = 0$. The unit vector $\hat{n}_{pl1}$ describes the direction of the normal to the plane. We assume that $\hat{n}_{pl1}$ lies in the y-z plane, points in the positive z direction when $\varphi = 0°$, in the positive y direction when $\varphi = 90°$, and tilts around the x axis at intermediate angles. This vector is:

$$\hat{n}_{pl1} = (0, -\sin\varphi, \cos\varphi)\,, \tag{94}$$

and the plane is then all the points (x, y, z) satisfying the equation:

$$-y\sin\varphi + z\cos\varphi = 0\,. \tag{95}$$

The force $\vec{\tau}$, that the root tip is applying on the agar plate due to gravitropism, is a result of a gravitropically induced bending of the FMZ, as discussed in the main text. The bending occurs in a plane that is perpendicular to the surface of the earth and that also includes the vector, $\hat{n}_{tip}$ that represents the projection of the direction at which the root tip is pointing to the agar surface. When the growth is in the downhill direction, $\hat{n}_{tip}$ (the green arrow in Fig 7 in the main text) is:

$$\hat{n}_{tip} = \hat{n}_{dh} = (0, -\cos\varphi, -\sin\varphi)\,. \tag{96}$$

When the growth direction forms an angle θ with respect to the downhill direction, this vector is rotated by an angle θ around a rotation axis in the direction of the normal to the growth plane $pl1$. The direction of the tilted growth vector can be found from Rodrigues' rotation formula:

$$\hat{n}_{tip} = \hat{n}_{dh}\cos\theta + (n_{pl1} \times \hat{n}_{dh})\sin\theta + \hat{n}_{pl1}(\hat{n}_{pl1}\cdot\hat{n}_{dh})(1-\cos\theta) \tag{97}$$

from which we get:

$$\hat{n}_{tip} = (\sin\theta, -\cos\theta\cos\varphi, -\cos\theta\sin\varphi)\,. \tag{98}$$

The plane in which the root curves includes $\hat{n}_{tip}$ and the vertical direction $\hat{z}$ and its normal vector is thus found using the vector product:

$$\vec{n}_{pl2} = \hat{z} \times \hat{n}_{tip} = (0,0,1) \times (\sin\theta, -\cos\theta\cos\varphi, -\cos\theta\sin\varphi) = (\cos\theta\cos\varphi, \sin\theta, 0)\,. \tag{99}$$

We can also write an equation for this plane:

$$\vec{n}_{pl2}\cdot\vec{r} = -x\cos\theta\cos\varphi + y\sin\theta = 0\,. \tag{100}$$

The direction at which the force $\vec{\tau}$ is applied by the root on the surface, is inside the plane $pl2$ and is perpendicular to the growth direction $\hat{n}_{tip}$. Its direction is the same as the direction of:

$$\vec{n}_{\tau} = \vec{n}_{pl2} \times \hat{n}_{tip} = (\cos\theta\cos\varphi, \sin\theta, 0) \times (\sin\theta, -\cos\theta\cos\varphi, -\cos\theta\sin\varphi) = \tag{101}$$

$$(-\cos\theta\sin\theta\sin\varphi,\ \cos^2\theta\cos\varphi\sin\varphi,\ -\cos^2\theta\cos^2\varphi - \sin^2\theta)\,. \tag{102}$$

We take into account the projection of this force into three components: a component pointing in the opposite direction to the normal to the growth plane $\hat{n}_N$ (into the growth surface) that gives rise to the normal force, a perpendicular component $\hat{n}_{dh}$ which lies in the growth plane and points towards the downhill direction, a component in the $\hat{x}$ direction that is positive when $\theta > 0$ and negative when $\theta < 0$ (Fig 7 in the main text). The magnitude of the component in the direction $\hat{n}_{dh}$:

$$\vec{n}_{\tau}\cdot\hat{n}_{dh} = \sin^2\theta\sin\varphi\,. \tag{103}$$

The magnitude of the component that is pointing in the growth plane in a direction orthogonal to n_{dh} (this is actually the x-direction) is:

$$\vec{n}_{\tau}\cdot\hat{x} = -\cos\theta\sin\theta\sin\varphi\,. \tag{104}$$

Both components are responsible for the symmetry breaking that leads to waving rather than coiling. Both are zero when the surface is horizontal ($\varphi = 0$) and when the root is growing in a downhill direction ($\theta = 0$), and for a finite φ, the first component causes the slip to have a downhill component when the static friction is overcome and the second acts as a retrieving force that counter-balances the change of direction of $\hat{n}_{tip}$ due to buckling. Together, these components keep an average downhill direction of the root tip when $\varphi > 0$.

[1] A. Goriely, *The mathematics and mechanics of biological growth*, Vol. 45 (Springer, 2017).

[2] J. L. Silverberg, R. D. Noar, M. S. Packer, M. J. Harrison, C. L. Henley, I. Cohen, and S. J. Gerbode, Proceedings of the National Academy of Sciences (2012).

[3] E. Kolb, V. Legué, and M.-B. Bogeat-Triboulot, Physical biology **14**, 065004 (2017).

[4] T. Kurokawa, T. Tominaga, Y. Katsuyama, R. Kuwabara, H. Furukawa, Y. Osada, and J. P. Gong, Langmuir **21**, 8643 (2005).
[5] L. D. Landau, E. M. Lifshitz, V. Berestetskii, and L. Pitaevskii, *Course of Theoretical Physics: Theory of Elasticity* (1995).
[6] C. Wang, International Journal of Non-Linear Mechanics **32**, 1115 (1997).
[7] L. Cedolin *et al.*, *Stability of structures: elastic, inelastic, fracture and damage theories* (World Scientific, 2010).
[8] M. V. Thompson and N. M. Holbrook, Plant Physiology **135**, 1822 (2004).
[9] N. G. De Bruijn, *Asymptotic methods in analysis*, Vol. 4 (Courier Corporation, 1981).
[10] J. Hutchinson, W. Koiter, *et al.*, Appl. Mech. Rev **23**, 1353 (1970).
