# Supplementary figures and images for: A mechano-sensing mechanism for waving in plant roots
Source: Sci Rep. 2022 Jun 10;12:9635. doi: 10.1038/s41598-022-14093-1 (PMC9187721; doi:10.1038/s41598-022-14093-1)

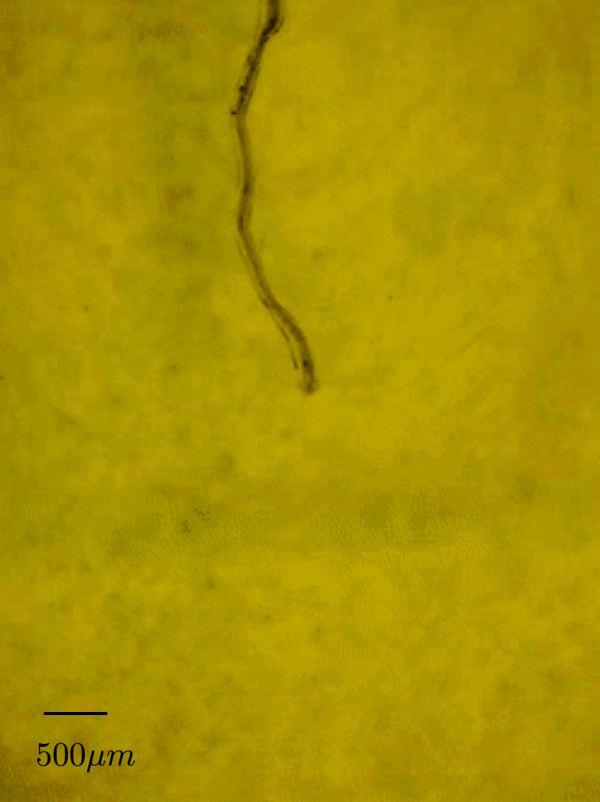

Supplement: Supplementary file 2 — Supplementary Movie 1. [file 41598_2022_14093_MOESM2_ESM.gif]

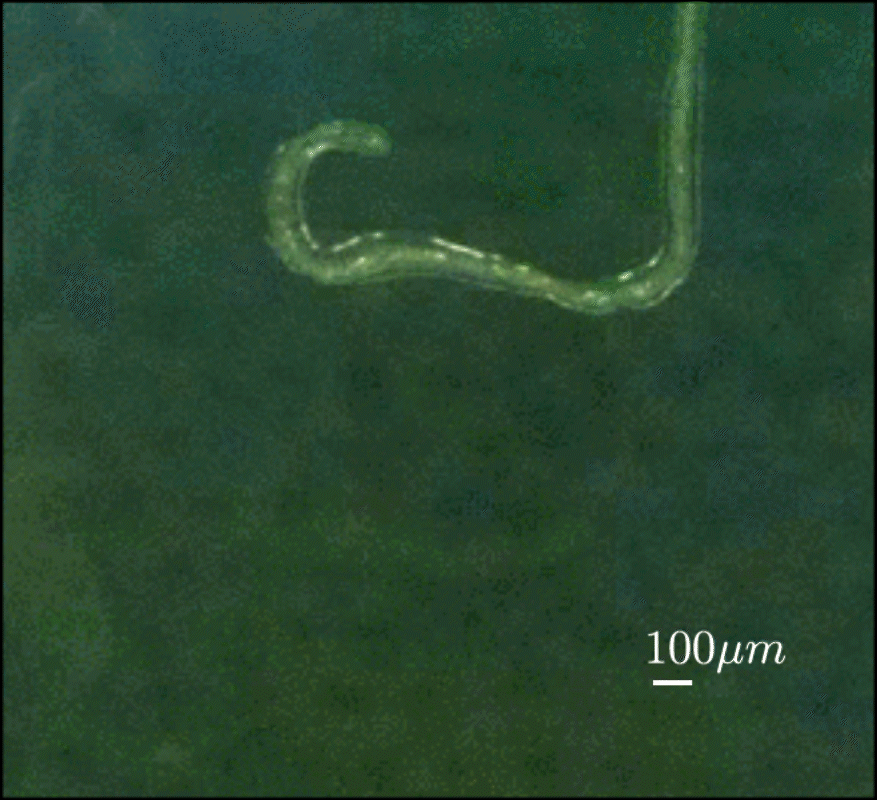

Supplement: Supplementary file 3 — Supplementary Movie 2. [file 41598_2022_14093_MOESM3_ESM.gif]

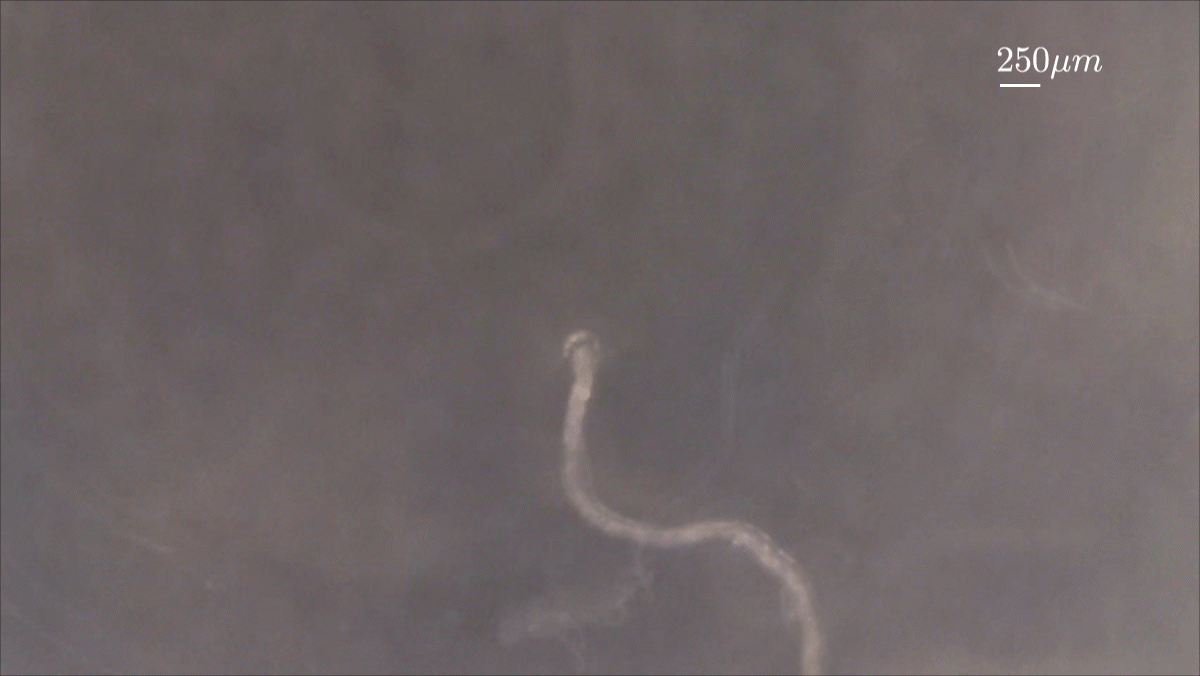

Supplement: Supplementary file 4 — Supplementary Movie 3. [file 41598_2022_14093_MOESM4_ESM.gif]
